# A unified model for the G1/S cell cycle transition

**DOI:** 10.1093/nar/gkaa1002

**Published:** 2020-11-09

**Authors:** Samuel Hume, Grigory L Dianov, Kristijan Ramadan

**Affiliations:** Medical Research Council Oxford Institute for Radiation Oncology, Department of Oncology, University of Oxford, Oxford OX3 7DQ, UK; Medical Research Council Oxford Institute for Radiation Oncology, Department of Oncology, University of Oxford, Oxford OX3 7DQ, UK; Institute of Cytology and Genetics, Siberian Branch of the Russian Academy of Sciences, Lavrentieva 10, 630090 Novosibirsk, Russian Federation; Novosibirsk State University, 630090 Novosibirsk, Russian Federation; Medical Research Council Oxford Institute for Radiation Oncology, Department of Oncology, University of Oxford, Oxford OX3 7DQ, UK

## Abstract

Efficient S phase entry is essential for development, tissue repair, and immune defences. However, hyperactive or expedited S phase entry causes replication stress, DNA damage and oncogenesis, highlighting the need for strict regulation. Recent paradigm shifts and conflicting reports demonstrate the requirement for a discussion of the G1/S transition literature. Here, we review the recent studies, and propose a unified model for the S phase entry decision. In this model, competition between mitogen and DNA damage signalling over the course of the mother cell cycle constitutes the predominant control mechanism for S phase entry of daughter cells. Mitogens and DNA damage have distinct sensing periods, giving rise to three Commitment Points for S phase entry (CP1-3). S phase entry is mitogen-independent in the daughter G1 phase, but remains sensitive to DNA damage, such as single strand breaks, the most frequently-occurring lesions that uniquely threaten DNA replication. To control CP1-3, dedicated hubs integrate the antagonistic mitogenic and DNA damage signals, regulating the stoichiometric cyclin: CDK inhibitor ratio for ultrasensitive control of CDK4/6 and CDK2. This unified model for the G1/S cell cycle transition combines the findings of decades of study, and provides an updated foundation for cell cycle research.

## INTRODUCTION

The decision to enter S phase is principally controlled by the cyclin-dependent kinases (CDKs), CDK4, CDK6 and CDK2. The three D-type cyclins, cyclin D1–3, associate with CDK4 and 6, forming cyclin D-CDK4/6 complexes. CDK4/6 activation leads to activation of CDK2 in turn, which, in G1 phase, pairs with the E-type cyclins, cyclin E1/2. Two families of CDK inhibitors, the inhibitors of CDK4 (INK4) and CDK interacting protein/kinase inhibitory protein (CIP/KIP) families, antagonise cyclins. The INK4 family members are p16^INK4A^, p15^INK4B^, p18^INK4C^ and p19^INK4D^, while the CIP/KIP family members are p21^Cip1^, p27^Kip1^ and p57^Kip2^. INK4 proteins only inhibit CDK4 and CDK6, while CIP/KIP proteins have broader specificity that enables their inhibition of CDK4, CDK6 and CDK2 (1–5).

Mitogens are the major stimulus for S phase entry. Mitogens include epidermal, fibroblast, and insulin growth factors (EGF, FGF and IGF), which bind their cognate cell surface receptors to activate intracellular signalling, including the mitogen-activated protein kinase (MAPK) pathway (6). c-Myc is a key transcription factor acting downstream of this pathway, whose activation stimulates S phase entry through regulation of cell cycle genes, including *CCND2* (encoding cyclin D2) (7).

DNA damage is the major inhibitor of S phase entry. DNA is an inherently unstable molecule (8), and each cell suffers ∼55 000 single strand breaks (SSBs) and ∼25 double strand breaks (DSBs) per day (9). A significant source of endogenous DNA damage is oxidative phosphorylation, which produces reactive oxygen species (ROS). Similarly, replication of DNA can result in the aberrant conversion of SSBs to DSBs, and the formation of DNA-protein crosslinks (DPCs) (9–11). In parallel, DNA is subjected to damage from exogenous sources, such as ultraviolet light and chemo/radio-therapies (12,13). ROS, SSBs and DSBs activate ataxia-telangiectasia mutated (ATM)-p53 signalling, repressing S phase entry through p53-dependent expression of the CDK inhibitor *CDKN1A* (which encodes p21) (14–18).

**Figure 1.**
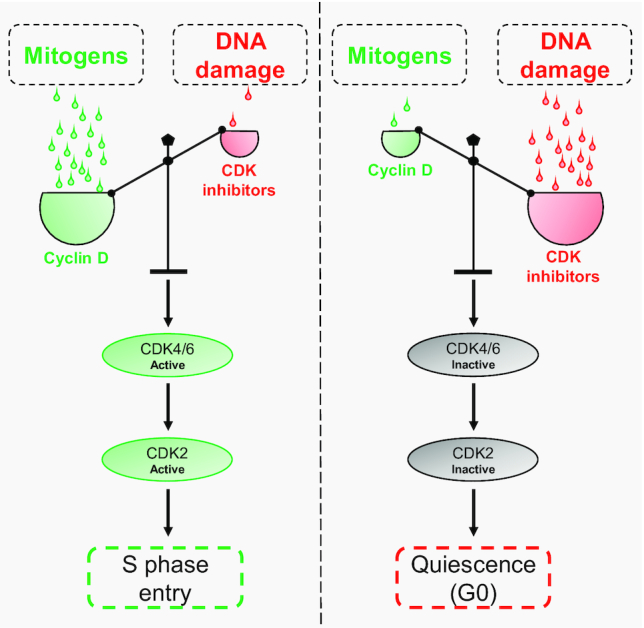
Mitogens and DNA damage compete to regulate the cyclin: CDK inhibitor balance and S phase entry. Where mitogens outcompete DNA damage, the stoichiometric balance of cyclin D: CDK inhibitors is shifted in favour of cyclin D, activating CDK4/6, leading to CDK2 activation, and driving S phase entry. In contrast, where DNA damage outcompetes mitogens, the balance is shifted in favour of the CDK inhibitors, resulting in CDK4/6 inhibition, inhibition of CDK2 in turn, and inducing quiescence.

Compelling experiments showed that increasing the concentration of mitogens in the presence of exogenous DNA damage proportionally increases DNA damage-resistant S phase entry (19). This leads to the fascinating conclusion that mitogens and DNA damage are in direct competition with one another to regulate S phase entry (19,20). Mechanistically, this competition controls the G1/S transition by regulating the balance of cyclins: CDK inhibitors, which are themselves in 1:1 stoichiometric competition for the regulation of CDKs (19,21). Consequently, where mitogens outcompete DNA damage, cyclins outcompete CDK inhibitors, CDKs are activated, and cells enter S phase. In contrast, where DNA damage outcompetes mitogens, CDK inhibitors outcompete cyclins, and cells enter quiescence (G0) (19–23) (Figure 1).

The classical model for the G1/S transition envisions a single ‘restriction point’ positioned towards the end of G1, after which cells are committed for entry into S phase. The early work that established this model mainly relied on an artificial experimental setup in which a population of hundreds of thousands of cells is released from synchronisation to a specific cell cycle phase (such as mitogen starvation-induced quiescence) to start the cell cycle (24–35). However, this approach can induce a stress response not triggered during physiological progression through the cell cycle, cannot observe cellular ancestors and cannot identify subpopulations of cells. In contrast, recent studies visualise fluorescent cell cycle sensors by single cell time-lapse microscopy (among other techniques) to tease apart S phase commitment with unprecedented intricacy (19–23,36–48). This method enables the precise analysis of actively-cycling individual cells, which organically progress through the cell cycle, undergoing mitosis to produce progenitors. Data from these ground-breaking studies have provided an entirely new perspective of the G1/S transition, and formed the foundation for a multitude of discoveries that complete the model.

Here, we discuss these findings, highlight conflicts, ask unanswered questions, and present a unified model for the S phase entry decision.

## MODELS FOR S PHASE COMMITMENT

### The classical model

The concept of an S phase commitment point (the restriction point) was first reported in 1974, as a stage in G1 after which cells enter—and irreversibly complete—S phase, independent of external stimuli (24). Cells passing the commitment point cannot return to quiescence, even if mitogen stimulation is discontinued or upon induction of DNA damage (24–32,34,35). This is essential to avoid incomplete DNA replication and resultant genomic instability (49–51).

The molecular basis of the commitment point was identified as hyperphosphorylation of Retinoblastoma protein (Rb), a cell cycle inhibitor phosphorylated on 14 CDK sites *in vivo* (30,31,33,34,49). Unphosphorylated Rb directly binds and inhibits the major G1/S transcription factors, E2F1, 2 and 3A (hereafter ‘E2Fs’), as well as inducing repressive chromatin at E2F-responsive promoters through recruitment of histone deacetylases (33,49,50). In the classical model, 100% of newly-born cells start G1 with unphosphorylated Rb, due to the action of protein phosphatase 1 (PP1) on hyperphosphorylated Rb from the previous cell cycle in mitosis (33,52,53). Subsequently, cumulative phosphorylation of Rb (hypophosphorylation), by cyclin D-CDK4/6 in early G1 phase, partially inhibits Rb and proportionally increases the activity of E2F (33). This permits E2F-dependent transcription of *CCNE1/2* (encoding cyclin E1/2) and consequent cyclin E-CDK2-dependent Rb phosphorylation (hyperphosphorylation) in late G1 (29,33). This completes Rb inactivation and enables full E2F-dependent transcription, engaging a self-sustaining positive feedback loop in which cyclin E-CDK2 maintains Rb hyperphosphorylation and E2F activity, while E2Fs synthesise new cyclin E molecules (25,29,30,33,34,49,50). Active E2Fs also stimulate the expression of a host of proteins that enable continued CDK2 activity in S phase, and promote the mechanics of DNA replication (34,51). These proteins include cyclin A2, the main S phase partner of CDK2, and ribonucleotide reductase family member 2 (RRM2), which synthesises deoxyribonucleotides required for replication (54). Rb hyperphosphorylation is maintained throughout the entire cell cycle, which is necessary to achieve irreversible cell cycle completion (34,49–52).

This mechanism for Rb inactivation, achieved in a stepwise manner through progressive hypophosphorylation by cyclin D-CDK4/6 followed by full hyperphosphorylation by cyclin E-CDK2, lacked explicit biochemical evidence, motivating a later paper that employed two-dimensional isoelectric focusing to detect Rb phosphorylation (31). In contrast to previous studies, this work proposed that cyclin D-CDK4/6 performs single phosphorylation on Rb in early G1 phase. This mono-phosphorylation did not result in partial inactivation of Rb, nor promote E2F activity, challenging the functional importance of Rb targeting by cyclin D-CDK4/6 in S phase entry. However, in agreement with the first studies, this report concluded that G1 cells are born with unphosphorylated Rb, and hyperphosphorylation of Rb by cyclin E-CDK2 in late G1 phase inactivates Rb to allow full E2F activity and S phase entry (31). These findings were later supported by others, using proteomics and phospho-specific antibodies (55).

**Figure 2.**
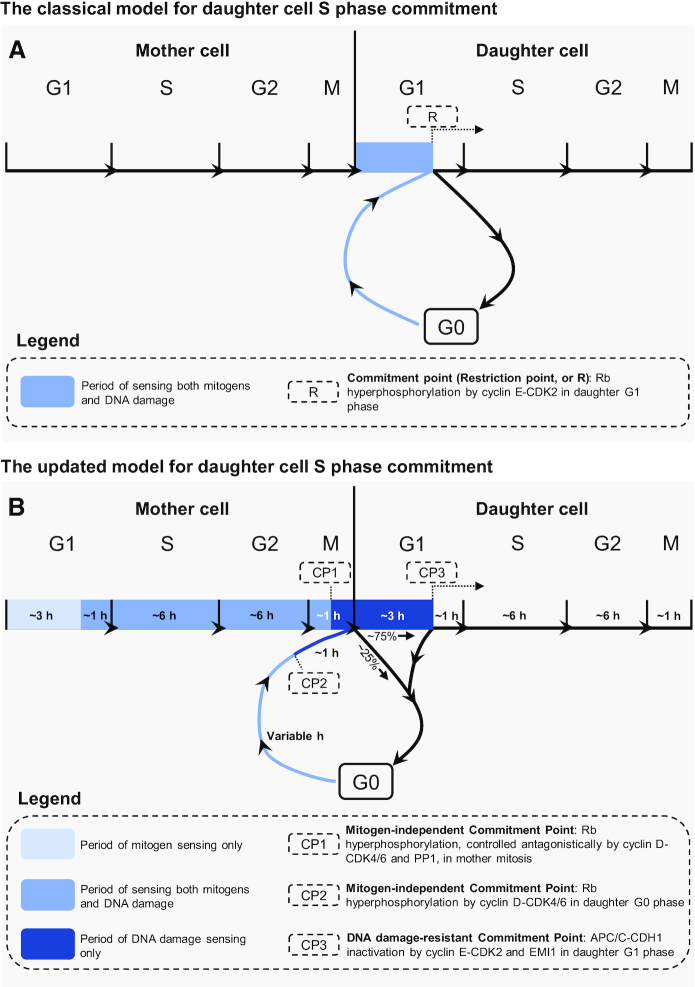
Classical and updated models for daughter cell S phase commitment. (**A**) In the classical model, there is one commitment point (the restriction point, or R), underpinned by cyclin E-CDK2-mediated Rb hyperphosphorylation and consequent inactivation in late G1 cells. In this model, all cells are born with unphosphorylated Rb, due to universal PP1-mediated dephosphorylation of Rb in the mother mitosis. Mitogens and DNA damage are only sensed in G1 phase (in cycling cells) or G0 (in cells emerging from quiescence), before Rb inactivation, to determine the irreversible S phase entry decision. (**B**) In the updated model, there are three distinct commitment points for the daughter G1/S transition. In this model, most cells are born with hyperphosphorylated Rb. The decision for Rb hyperphosphorylation occurs in the mother mitosis, at CP1, which is controlled through sensing of mitogens and DNA damage during the mother cell cycle. Cells successfully passing CP1 transmit hyperphosphorylated Rb to the daughter G1, and cyclin D-CDK4/6 is exclusively responsible for maintaining Rb hyperphosphorylation throughout the daughter G1. CP2 is for cells that are unable to pass CP1; these cells dephosphorylate Rb in mitosis and leave the cell cycle for G0. To pass CP2, cells sense mitogens and DNA damage in G0 to control inactivation of Rb, after which point they can progress into G1. For CP3, the point-of-no-return for S phase entry, there is an additional period of DNA damage sensing, stretching from Rb inactivation at CP1/2 to APC/C-CDH1 inactivation.

Despite uncertainties about how cells arrive at Rb hyperphosphorylation, these reports laid the ground for the classical model, suggesting that inactivation of Rb, by cyclin E-CDK2 in late G1 phase, forms the molecular basis for S phase commitment. In this model—based predominantly on cell cycle release from quiescence—loss of mitogens or induction of DNA damage, during a sensing period between cell birth and Rb inactivation, can reverse S phase entry and direct cells to quiescence (24–28,30–32,34,35) (Figure 2A).

### The updated model: three distinct S phase commitment points

Subsequent technological advances enabling the study of actively-cycling single cells lead to new findings that overturn the classical model, most prominently concerning multiple aspects of the mechanisms that control Rb. Firstly, new research has shown that the majority of cells are born with hyperphosphorylated Rb, which is inherited from the previous cell cycle. Contrasting the original model, only a small fraction of cells is subject to PP1-mediated Rb dephosphorylation in mitosis (19,22,23,36,40,42,48). Secondly, experiments using CDK4/6 and CDK2 inhibitors to examine Rb phosphorylation in single cells argued that cyclin E-CDK2 does not contribute to Rb hyperphosphorylation in G1 cells (47). Instead, cyclin D-CDK4/6, which (in most cells) maintains hyperphosphorylation of Rb from the mother mitosis, is sufficient for Rb hyperphosphorylation and inactivation throughout the daughter G1, while cyclin E-CDK2 (and cyclin A2-CDK2) are only responsible for sustaining Rb hyperphosphorylation after the start of S phase (36,47,48). Finally, elegant experiments convincingly confirmed that Rb targeting by cyclin D-CDK4/6 plays a major role at the G1/S transition: mutating the binding site for cyclin D-CDK4/6 on Rb prevents cyclin D-CDK4/6-mediated Rb phosphorylation, impedes Rb inactivation, inhibits S phase entry, and stops tumourigenesis (56).

The fact that most daughter cells are born with hyperphosphorylated Rb suggests that they are born committed for S phase entry. Indeed, pioneering work employing time-lapse microscopy demonstrated that removal of mitogens or inhibition of MAPK signalling during G1 phase of daughter cells, before the conceptual position of the restriction point identified by the classical model, does not disrupt S phase entry (22). Instead, mitogens must be removed before Rb hyperphosphorylation in mitosis of the mother cell cycle to repress the daughter G1/S transition (19,22,42,48,57). Remarkably, loss of mitogen signalling for as little as an hour, even in early G1 phase of the mother cell cycle, prevents Rb hyperphosphorylation in mitosis and reduces S phase entry of daughters (19,22,23,36,42,48) (Figure 2B).

In parallel, other papers employing similar single-cell approaches in cycling cells showed that, analogous to mitogens, DNA damage occurring during the mother cell cycle can block daughter cells’ S phase entry. This DNA damage mainly arises from DNA replication stress, and results in Rb dephosphorylation in mitosis (19,22,23,40,45). Therefore, these reports inverted the original model that mitogens and DNA damage are exclusively sensed in G1 phase of daughter cells, showing that the sensing periods for mitogens and DNA damage extend into cellular ancestors (Figure 2B).

Intriguingly, although cells no longer require mitogens after Rb hyperphosphorylation in the mother mitosis, landmark results revealed that induction of DNA damage can still halt S phase entry and return cells to quiescence after Rb inactivation (42). This suggests that Rb inactivation designates mitogen-independent—but not DNA damage-resistant—cell cycle commitment. Specifically, DNA damage occurring in daughter G1 cells can induce quiescence up to a defined timepoint, ∼1 h before S phase (36,42,43) (Figure 2B).

This leads to the conclusion that DNA damage sensing continues beyond mitogen sensing, and that there is a novel molecular basis for DNA damage-resistant S phase commitment. A promising candidate for this molecular basis is the inactivation of anaphase-promoting complex/cyclosome with the substrate adapter protein CDH1 (APC/C-CDH1) (42,43). APC/C-CDH1 is a ubiquitin ligase complex that directs the degradation of many S phase proteins in early G1 phase, including cyclin A2 and RRM2 (58). Indeed, by combining measurements of Rb hyperphosphorylation, a sensor for APC/C-CDH1 activity, and single cell time-lapse microscopy, revolutionary studies showed that DNA damage returns cells to quiescence up to—but not after—APC/C-CDH1 inactivation. APC/C-CDH1 inactivation occurs substantially later (∼4 h) than Rb inactivation, and there is a short and consistent time gap (∼1 h) between APC/C-CDH1 inactivation and irreversible S phase onset (36,42,43) (Figure 2B).

Taken together, these findings establish a rationale for three distinct S phase commitment points. Because cycling cells lose their requirement for mitogens after Rb hyperphosphorylation in mitosis, the first commitment point for S phase entry is a mitogen-independent commitment point, underpinned by Rb inactivation in the mother cell mitosis (22,23,36,40,42,48). This commitment point is termed CP1. Although passage through CP1 confers mitogen-independence, DNA damage occurring in the mother cell cycle also disrupts Rb hyperphosphorylation and daughter cell S phase entry, suggesting that CP1 also receives substantial input from DNA damage (19,22,23,40,45). The ability of cells to maintain hyperphosphorylation of Rb at CP1 forms the molecular basis for an unprecedented bifurcation into two populations as cells leave mitosis (19,22,23,36,40,42,48). In the MCF10A cell line (an immortalised non-cancer human mammary epithelial cell line), most cells (∼75% of physiologically-cycling cells), which experience a cell cycle with sufficient mitogen provision and limited replication stress-induced DNA damage, successfully maintain Rb hyperphosphorylation in mitosis and pass CP1, proceeding to G1 phase in daughter cells (19,22,23,36,40,42,48). The remaining cells (∼25% of physiologically-cycling cells), which experience mitogen disruption or replication stress-mediated DNA damage during their cell cycle, undergo Rb dephosphorylation in mitosis, failing passage through CP1. This population can progress through mitosis, but then exits the cell cycle to quiescence, with dephosphorylated Rb (19,22,23,36,40,42,45,48,52). As a result, this population regains sensitivity to both mitogens and DNA damage, and must re-attempt Rb hyperphosphorylation, requiring a second commitment point after which cells are mitogen-independent, CP2, that is positioned at the end of G0 phase (19,22,36,42). Finally, since cells can still halt S phase entry and return to quiescence upon induction of DNA damage after inhibition of Rb—but only up to inactivation of APC/C-CDH1—a third commitment point must exist after passage through CP1 or CP2, which underlies DNA damage-resistant S phase entry, is positioned at the end of G1 phase, and is underpinned by APC/C-CDH1 inactivation (36,42,43). This final commitment point is termed CP3 (Figure 2B).

In these ways, the original model has been uprooted in favour of a new model with three distinct S phase commitment points: CP1, CP2 and CP3. How are these commitment points controlled, and what are the details of their molecular basis?

### Molecular basis for commitment point 1 (CP1)

**Figure 3.**
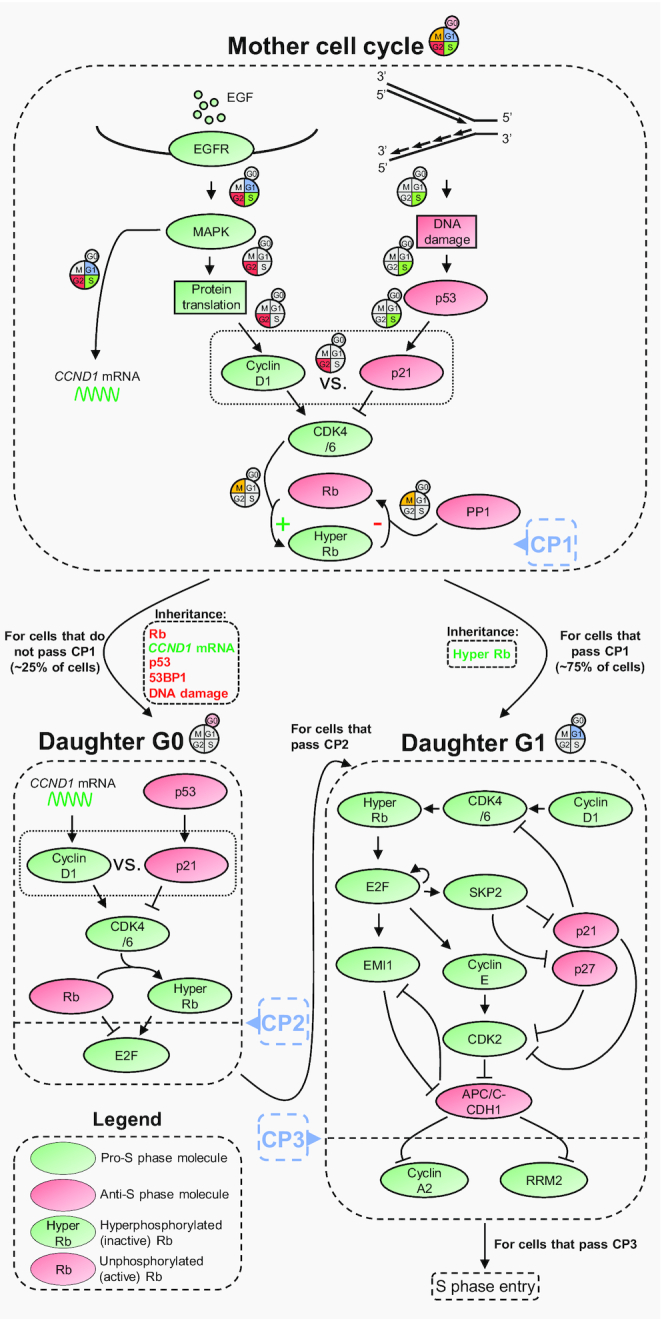
Molecular basis for the S phase commitment points. Rb hyperphosphorylation at CP1 is controlled by competition between CDK4/6-mediated Rb phosphorylation and PP1-driven Rb dephosphorylation. CDK4/6 is regulated by the stoichiometric competition of cyclin D1 and p21, which sense mitogens and DNA damage, respectively. PP1 is also likely subject to antagonistic regulation by mitogens and DNA damage, through cyclin B-CDK1 and p21 (not shown in the figure). Most cells maintain Rb hyperphosphorylation for successful passage through CP1, and hyperphosphorylated Rb is transmitted as part of an inheritance package to the daughter G1 phase, relieving inhibition of E2F and triggering feedback loops that lead to inactivation of APC/C-CDH1 and passage through CP3. Unhealthy cells dephosphorylate Rb during mitosis and leave the cell cycle for G0. These cells can re-enter the cell cycle if they encounter more mitogens than DNA damage, but are also susceptible to the mitogen and DNA damage history of their mothers through *CCND1* mRNA, p53, 53BP1, and DNA damage itself. These factors co-operate to regulate CDK4/6 activity for Rb hyperphosphorylation, and, in turn, CP2. Coloured segments indicate the stage of the cell cycle where each event occurs.

CDK activity maintains Rb hyperphosphorylation throughout the entire cell cycle, and the decision between dephosphorylation and hyperphosphorylation occurs in mitosis of mother cells, at CP1 (19,22,23,36,40,42,45,48,52) (Figure 2B). Rb hyperphosphorylation in mitosis is regulated through two primary mechanisms. The first depends on phosphorylation of Rb by CDK4/6, itself regulated through the stoichiometric competition between cyclin D1 and p21 in G2 phase (19,22,23,36,40,45,48). The second mechanism is less well-characterised, but depends on PP1-mediated Rb dephosphorylation, itself likely subjected to inverse regulation by cyclin B-CDK1 and p21 during mitosis (22,23,52,53,59,60) (Figure 3).

Cyclin D1 and p21 are uniquely suited to regulate CDK4/6 activity during the mother G2 (19,22,23,36,40,45,48). Cyclin D1’s half-life is very short (∼30 min in cells treated with the translation inhibitor cycloheximide) (19), rendering the protein exquisitely sensitive to translation rates: increased or decreased translation is followed by increased or decreased cyclin D1 levels, respectively. Mechanistically, MAPK signalling connects mitogen provision to the translation machinery, regulating translation rates depending on the mitogen load (48). Given sufficient mitogens, translation rates continuously increase throughout the cell cycle, leading to upregulation of cyclin D1 in G2 phase. In contrast, even a transient drop in mitogens, at any point before mitosis of the mother cell cycle, reduces MAPK signalling and lowers the translation rate in G2. Therefore, the mother cell cycle senses mitogens, transmitting this information to cyclin D1 levels in G2 phase (48) (Figure 3).

p21 is mainly produced during the mother S phase, driven by replication stress-induced DNA damage, which mediates p53-dependent expression of the *CDKN1A* mRNA (19,22,40,45). Notably, although this induces immediate C*DKN1A* mRNA upregulation, p21 protein levels do not increase until the mother G2 phase. This is due to the high activity p21-directed ubiquitin ligases during S phase, which coordinate p21’s polyubiquitination and consequent proteasome-dependent degradation, preventing interference with ongoing replication in a context of mild endogenous DNA damage (20,45,61,62). Therefore, the mother cell cycle senses DNA damage, transmitting this information to p21 levels in G2 phase (22,40,45) (Figure 3).

Consequently, where mitogens outcompete DNA damage during the mother cell cycle, cyclin D1 outweighs p21 in G2 phase and CDK4/6 is active, promoting Rb phosphorylation in mitosis. Conversely, an increased ratio of DNA damage: mitogen provision results in out-competition of cyclin D1 by p21 in G2, rendering CDK4/6 inactive and reducing Rb phosphorylation (19,22,23,36,40,42,45,48) (Figure 3).

As well as repressing the CDK4/6-mediated phosphorylation of Rb, elegant work demonstrated that upregulation of p21, produced after replication stress during the mother cell cycle, also induces dephosphorylation of Rb, which occurs in mitosis (23). This dephosphorylation is likely performed by PP1, the major mitotic phosphatase for Rb (23,52,53). Although molecular details for p21-induced Rb dephosphorylation are not known, a major regulatory mechanism for PP1 during mitosis is cyclin B-CDK1-mediated phosphorylation of the PP1-α catalytic subunit at T320, which inhibits PP1 (23,59,60). Since p21 inhibits cyclin B-CDK1 in turn, via multiple mechanisms (63–67), it is possible that p21-mediated CDK1 inhibition results in activation of PP1 for Rb dephosphorylation in the mother cell mitosis. In parallel, other mechanisms could also contribute. For example, DNA damage-mediated activation of PP1 through dissociation of PP1 from its inhibitory interaction partner PNUTS (68,69) or upregulation of PP1 by DNA damage (70) may be important. However, these pathways are p21-independent, and lack the temporal control needed for a mechanism of Rb dephosphorylation executed precisely in mitosis (23). Therefore, given that CDK1 only inhibits PP1 during mitosis (60), a putative p21-CDK1-PP1-Rb axis may be the best candidate for regulation of Rb dephosphorylation downstream of p21.

Like the cyclin D1-p21 stoichiometric competition senses both DNA damage and mitogens to regulate Rb phosphorylation through CDK4/6, it is likely that the mechanism for Rb dephosphorylation by PP1 is also mitogen-responsive. For example, *CCNB1* (encoding cyclin B1) is under direct positive transcriptional control of c-Myc, itself a sensitive reader of mitogens (71). As such, we envision that competition between cyclin B and p21, which sense mitogens and DNA damage, respectively, regulates Rb dephosphorylation through control of CDK1 and, consequently, PP1. Despite lack of activity of cyclin B-CDK1 in G1 cells, this would additionally implicate cyclin B-CDK1, by repressing Rb dephosphorylation at CP1, in an important role at the daughter G1/S transition, which warrants further investigation.

Intriguingly, the binding sites for cyclin-CDKs and PP1 on Rb are overlapping and mutually exclusive (72). Because of this, we propose that, ultimately, it is competition between CDK4/6-mediated phosphorylation and PP1-mediated dephosphorylation that controls whether Rb is hyperphosphorylated at CP1. Where mitogens are plentiful and replication stress limited, CDK4/6 wins, Rb is hyperphosphorylated, and cells successfully pass CP1 (∼75% of cells). However, where mitogens are limited or cells experience replication stress, PP1 wins, Rb is dephosphorylated, and cells fail to pass CP1 (∼25% of cells) (19,22,23,36,40,42,45,48) (Figure 3).

Cells failing to pass CP1 due to endogenous replication stress emerge as daughter G0 cells. This means that endogenous DNA damage occurring during the mother S phase upregulates p21 in G2 and causes Rb dephosphorylation in mitosis, but permits cell division (19,22,23,40,45). This is an unexpected finding, since induction of exogenous DNA damage during the mother S phase, for example through radiomimetics or topoisomerase II inhibition, induces arrest in the mother G2 (73,74). Therefore, why endogenous DNA damage allows cells to divide is a key question, especially since mitosis can exacerbate DNA damage, induce genomic instability, and cause cell death by mitotic catastrophe (75). Answering this question from a mechanistic perspective, endogenous replication stress induces much lower levels of p21 than exogenous DNA-damaging agents, levels that are insufficient to overpower the very high combined activity of CDK1 and CDK2 in G2 required for activation of the G2/M checkpoint (9,23,75). Furthermore, Rb dephosphorylation does not prevent mitotic progression, where Rb has little role (49,50). In mitosis, DNA damage arising from mild replication stress can undergo repair (76), and very few cells with endogenous replication stress die from mitotic catastrophe as a result (40,45,77). Finally, residual DNA damage can also be repaired in the daughter G0/G1 phase, providing a second opportunity to preserve genomic integrity (40,45,78,79).

The temporal position of CP1 in mother cells, and subsequent bifurcation into two distinct populations controlled by Rb hyperphosphorylation, was discovered in MCF10A cells, an immortalised epithelial cell line that does not express the major CDK inhibitors p15 or p16 (22). Although still the subject of disagreement (46), this phenomenon is reproducible in other cell lines. These include MCF10A cells in which p16 expression is rescued (36), other immortalised epithelial cells that do express p15/p16 (RPE1-hTERT), as well as primary fibroblasts (HLF) and even some cancer cells (U2OS, MCF7) (23). However, CP1 depends on Rb, and loss of Rb function in many cancer cells (including HeLa) abolishes CP1, permitting S phase entry even in the absence of mitogens or presence of DNA damage (39,41). Notably, although the role of endogenous replication stress during the mother S phase provides well-established control of the daughter G1/S transition (19,22,23,40,45), a parallel contribution from ROS-mediated DNA damage arising from mother cell respiration has not been studied (to our knowledge), and may be an important avenue for future research.

### Molecular basis for commitment point 2 (CP2)

The ∼25% of cells that fail to hyperphosphorylate Rb at CP1 inherit dephosphorylated Rb, and are born in G0. To enter S phase, these cells must re-attempt Rb hyperphosphorylation, giving rise to a second mitogen-independent commitment point in the daughter G0 phase, CP2. Similar to CP1, if the balance of new mitogen provision is greater than the new DNA damage load, Rb hyperphosphorylation at CP2 can be mediated by cyclin D-CDK4/6 (19,22,36,42,47) (Figure 2B).

Nevertheless, daughter G0 cells continue to be influenced by their mothers for CP2. Although the cyclin D1 protein is short-lived (half-life ∼30 min), its mRNA, *CCND1*, has a much longer half-life (>3 h). Similarly, p21 is short-lived (half-life ∼1 h), but the half-life of p53 is over 7 h in conditions of DNA damage (increasing from ∼30 min under basal conditions), due to ATM-dependent inhibition of its ubiquitin ligase, MDM2 (19,80). These extended half-lives facilitate the transmission of *CCND1* and p53 from mothers to daughters (19). In daughter cells, *CCND1* mRNA is translated, forming cyclin D1 protein, and p53 transcribes *CDKN1A*, which produces p21 protein. The resulting stoichiometric competition between cyclin D1 and p21 contributes to the regulation of CDK4/6 activity and Rb phosphorylation for CP2 (19,45) (Figure 3).

Mother cells also transmit DNA damage itself, and associated DNA damage response (DDR) molecules, to influence CP2. For example, p53-binding protein 1 (53BP1) is an essential DSB repair factor that is among the first proteins recruited to DSBs, acting as a suppressor of DNA end-resection to direct DSB repair via non-homologous end joining (NHEJ) (81). DNA damage with bound 53BP1 is directly transmitted to daughter cells from mothers exposed to replication stress, leading to an elongated G0/G1 in daughters and generating more time for DNA repair before S phase (40,78,79). Mechanistically, as well as its role in DNA repair, 53BP1 promotes p53 signalling, by coordinating ubiquitin-specific peptidase 28 (USP28)-mediated p53 deubiquitination. This leads to p53 stabilisation and consequent C*DKN1A e*xpression (82,83). Therefore, increased 53BP1 inheritance increases the length of G0/G1 by shifting the cyclin D1: p21 balance in favour of p21, inhibiting passage through CP2 (Figure 3).

Subsequent research lead to the surprising finding that the inheritance of DNA damage and DDR molecules following replication stress in mother cells is not symmetric (84). This study induced replication stress through treatment with aphidicolin or overexpression of c-Myc, and found that one daughter cell inherits the majority of the damage, reducing its ability to enter S phase and increasing its time spent in G0. In contrast, the sister cell inherits clean DNA, increasing its chance for S phase entry. This mechanism could enable the sustained proliferation of a population of cells experiencing replication stress, but may be hijacked in cancer as a result (84). It should be noted that other studies found that sister cells tend to agree with one another on the S phase entry decision following endogenous replication stress in mother cells (22,41), suggesting that the mechanism identified by Xing *et al.* may be specific to exogenous (or high levels of) replication stress.

In summary, cells that successfully proceed through CP1 inherit hyperphosphorylated Rb and have a short, consistent G1 length. However, cells unable to traverse CP1 enter G0, inheriting unphosphorylated Rb. These cells can persist in G0, or can transition through CP2, depending on new mitogen and DNA damage information, as well as historical signalling from mother cells. This transition can be immediate, or can take several hours (or even days), explaining findings that G0/G1 length is the most flexible in the cell cycle (19,22,23,36,40,42,79,85) (Figure 3).

### Molecular basis for commitment point 3 (CP3)

Despite the overbearing influence of their mothers, the final decision for S phase entry lies with daughter cells themselves due to CP3, an emergency commitment point which exclusively responds to DNA damage, and is underpinned by inactivation of APC/C-CDH1. After cells successfully pass CP3, S phase entry is irreversible and will occur even upon DNA damage (36,42,43). Accordingly, we term CP3 the DNA damage-resistant commitment point (Figure 2B).

The mechanism for APC/C-CDH1 inactivation at CP3 involves sequential steps and is heavily dependent on E2F (43). Activation of E2F through CDK4/6-mediated inhibition of Rb increases levels of cyclin E1/2 and activates CDK2 (29,36,47). This enables CDK2-mediated phosphorylation and partial inhibition of APC/C-CDH1. As a result, APC/C-CDH1-directed degradation of early mitotic inhibitor 1 (EMI1), which is both a degradation substrate and inhibitor of APC/C-CDH1, is prevented (43). In parallel, active E2Fs transcriptionally induce *FBXO5* (the gene encoding EMI1), potentiating EMI1’s accumulation and allowing EMI1 to complete APC/C-CDH1 inactivation by preventing the interaction between APC/C-CDH1 and its E2 ubiquitin conjugating enzymes (43,86). In another upheaval of the classical model, this means that the major role for CDK2 at the G1/S transition is inactivating APC/C-CDH1, not Rb (36,42,43,47) (Figure 3). The inactivation of APC/C-CDH1 is reinforced through the ubiquitin-dependent degradation of CDH1 in S phase by the SKP1-Cullin-F-box (SCF) family ubiquitin ligase, SCF-cyclin F (87), as well as through deubiquitination of the critical APC/C-CDH1 target cyclin A2 in late G1 and S phases by the E2F-induced deubiquitinase USP37 (88). Consistent with a major inhibitory role at the G1/S transition, APC/C-CDH1 has recently been proposed as a constituent of a ‘brake model’ for cell cycle progression, pressure on which must be released to permit S phase entry (89).

In addition to E2Fs’ requirement for APC/C-CDH1 inactivation, E2Fs share many transcriptional targets with APC/C-CDH1’s degradation targets. For example, the upregulation of cyclin A2, RRM2 and EMI1 at the G1/S transition is dependent on the combined action of E2F-dependent transcription and loss of APC/C-CDH1-dependent degradation (43,54,58). Therefore, although Rb inactivation and APC/C-CDH1 inactivation form distinct commitment points, they are inextricably linked. E2Fs and APC/C-CDH1 do not act independently, and it is their co-ordinated function that ensures robust control of S phase entry.

It should be noted that other models favour the proteasomal degradation of p21, which also occurs in late G1 at a similar time to APC/C-CDH1 inactivation, as the identity of the final commitment point for S phase entry (20,90). Furthermore, in cells treated with CDK4/6 inhibitors, Rb inactivation becomes the final, DNA damage-resistant commitment point, suggesting that the molecular basis for cell cycle commitment is adaptable, not rigid (41).

In summary, three commitment points control entry to S phase: CP1, the mitogen-independent commitment point at the mother mitosis; CP2, the mitogen-independent commitment point in the daughter G0; CP3, the DNA damage-resistant commitment point, at the end of the daughter G1 (Figures 2B and 3).

## IRREVERSIBILITY OF THE G1/S TRANSITION: THE ROLE FOR FEEDBACK LOOPS

After committing to the cell cycle, cells must irreversibly complete S phase to avoid incomplete replication and consequent genomic instability. This relies on stimulus-independent inactivation of Rb and APC/C-CDH1, both of which are bistable switches that maintain their own inhibition through feedback loops (34,39,42,43,47).

Rb hyperphosphorylation is sustained throughout G1—independent of mitogens—by cyclin D-CDK4/6. Mechanistically, this is driven through a slow loss of cyclin D1 if mitogens are deprived that (mostly) maintains CDK4/6 activity until the end of G1 (47). Responsible for this is the long stability of the *CCND1* mRNA, which means that cyclin D1’s half-life in cells deprived of mitogens is ∼seven times longer than in cells treated with the translation inhibitor cycloheximide (19,47). This ensures that cells reach S phase, at which point Rb hyperphosphorylation is independently sustained through a positive feedback loop between Rb-E2F and cyclin E/A2-CDK2, which take over from cyclin D-CDK4/6 (47). In addition, E2Fs direct their own transcription, providing a second positive feedback loop (34,46,91). Furthermore, a third, dual-negative feedback loop between APC/C-CDH1 and EMI1 sustains the inactivation of APC/C-CDH1, even upon induction of DNA damage (39,43). Finally, these mechanisms are subject to fundamental reinforcement through degradation of the CDK inhibitors, p21, p27 and p57, ensuring high CDK2 activity during the G1/S transition and throughout S phase. This is directed by the SCF-SKP2 complex (for p21, p27 and p57) (39,61,92,93) and the Cullin-RING ubiquitin ligase (CRL) family member CRL4-CTD2 (for p21) (20,45,62). Since SKP2 is a transcriptional target of E2F and a degradation target for APC/C-CDH1, this supports a fourth feedback loop (39,94–97) (Figure 3). Therefore, intricate and intertwining feedback loops are central to the irreversibility of the G1/S transition, acting to sustain CDK- and EMI1-dependent inactivation of Rb and APC/C-CDH1, from the onset of their inactivation and throughout S phase.

## DNA DAMAGE SENSING BETWEEN CP1/2 AND CP3

**Figure 4.**
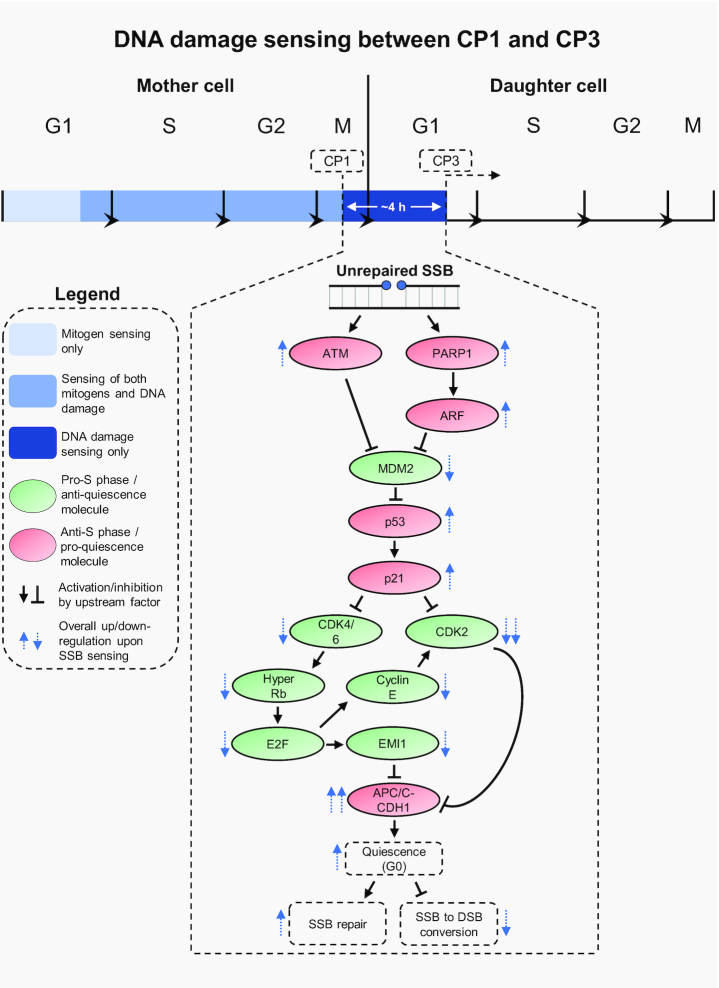
DNA damage sensing between CP1 and CP3. For the irreversible S phase commitment decision, mitogens are only sensed up to CP1 (in cycling cells, or up to CP2 in cells emerging from quiescence) while DNA damage is sensed up to CP3, demonstrating that DNA damage sensing extends beyond mitogen sensing. SSBs are the most frequently-occurring endogenous lesions, and signalling of unrepaired SSBs is most urgent in G1, before their aberrant conversion to DSBs by DNA replication. Therefore, we propose that cells employ specialised pathways to signal unrepaired SSBs that occur between CP1 (or CP2) and CP3. The major pathways, which are dependent on ATM and PARP1, aim to instigate p53-dependent quiescence, preventing S phase entry by reversing Rb hyperphosphorylation and stopping inactivation of APC/C-CDH1. Ultimately, these pathways repress SSB to DSB conversion in S phase, promote SSB repair, and preserve genome stability.

It is compelling that DNA damage-resistant commitment to S phase (at CP3), occurs much later (∼4 h) than mitogen-independent S phase commitment (at CP1/CP2), and indicates that an extended period of time, mainly in the daughter G1 phase, is dedicated to DNA damage sensing (36,42) (Figure 4). This raises important questions: which are the major lesions sensed during this extended period? And how do such lesions prevent inactivation of APC/C-CDH1 and return cells to quiescence after Rb inactivation?

DSBs are a potent type of DNA damage whose formation in G1 phase, prior to APC/C-CDH1 inactivation, strongly induce quiescence (42). Although DSBs undoubtedly contribute to lesion sensing between CP1/2 and CP3, formation of endogenous DSBs is relatively rare, occurring at a frequency of ∼25 per cell per day. In contrast, SSBs occur at the highest rate of any endogenous lesion (∼75% of all lesions: ∼55 000 per cell per day) (9). Furthermore, unrepaired SSBs represent the lesions that require the most urgent sensing to halt S phase entry, since collision of the CDC45-MCM2–7-GINS (CMG) helicase with SSBs during DNA replication collapses replication forks and produces DSBs (15,98). This can lead to cell death, as well as genomic rearrangements and cancer (9). Strikingly, fork collapse is unique to CMG’s collision with SSBs, and does not occur with other DNA adducts (98). For these reasons, we propose that unrepaired SSBs are particularly pertinent lesions for the extended period dedicated to DNA damage sensing.

Indeed, cells have evolved highly specialised pathways to signal SSBs that escape repair coordinated by the SSB repair scaffold protein, XRCC1, in G1 cells (11). These pathways are directed by the major DDR kinase, ATM (15), and the SSB sensor, poly(ADP-ribose) (PAR) polymerase 1 (PARP1) (99).

ATM is best known for its activation by DSBs and oxidative stress (16,17). However, studies specifically inducing SSBs, through low doses of methyl methanesulfonate (MMS) or siRNA-mediated depletion of XRCC1, lead to a paradigm shift, showing that unrepaired SSBs are sufficient for activation of ATM (15). This is a fundamental function of ATM that allows sensing of unrepaired SSBs in G1 phase. Consequently, unrepaired SSBs activate the ATM-p53-p21 pathway and, as a result, loss of ATM or p53 upon SSB induction causes unscheduled entry into S phase, replication over SSBs and catastrophic DSB formation (15) (Figure 4).

In addition, PARP1 recognises, binds to, and protects unrepaired SSBs, using nicotinamide adenine dinucleotide (NAD+) to polymerise chains of PAR, decorating itself and numerous substrates at the break site to promote repair (11). As a result, PARP1 activity at unrepaired SSBs consumes NAD+. If subsequent XRCC1-dependent repair is unsuccessful at the first attempt, successive PARP1 molecules bind, prompting further NAD+ consumption, and multiple such cycles exhaust the cellular NAD+ pool (100). This triggers a SIRT1-E2F1 (101)-p14^ARF^ (102)-MDM2-p53-p21 (103) cascade that promptly halts G1 progression and prevents S phase entry (99) (Figure 4). Importantly, combined PARP1 and ATM loss causes DSB formation and elicits synthetic lethality, which could reflect their importance in acting redundantly to prevent entry of SSB-containing cells into S phase (104).

Therefore, signalling of unrepaired SSBs via ATM and PARP1 activates p53 and prevents conversion of SSBs to DSBs in S phase. How would such DNA damage, occurring between CP1/2 and CP3, prevent inactivation of APC/C-CDH1 within the ∼4 h gap after Rb hyperphosphorylation, in order to stop S phase entry? Inactivation of APC/C-CDH1 relies on CDK2 and EMI1 (43), suggesting that pathways to disrupt its inactivation would target these factors. Critically, Rb hyperphosphorylation, which is upstream of CDK2 and EMI1, is self-sustaining in the absence of mitogens, but reversible if cells encounter DNA damage (36,42). How is this reversal achieved? p53 activation upregulates p21 within ∼2 h of DNA damage, inhibiting cyclin D-CDK4/6 (14,18) and, since continuous cyclin D-CDK4/6 activity is required to maintain Rb hyperphosphorylation throughout G1 (47), cyclin D-CDK4/6 inhibition by p21 would acutely repress Rb hyperphosphorylation. This would, in turn, abrogate E2F activity, reducing cyclin E1/2 and EMI1 levels. Furthermore, the presence of p21, coupled with the absence of cyclin E, would drive dual inhibition of CDK2, and this, combined with the lack of EMI1, would ultimately prevent inactivation of APC/C-CDH1, inducing quiescence (36,42,43) (Figure 4).

In addition to these pathways, it is likely that others, such as direct Rb dephosphorylation (52,105) or ATM-mediated cyclin D1 degradation (106,107) contribute to preventing APC/C-CDH1 inactivation upon DNA damage after Rb inhibition in G1 cells. Interestingly, early G1 cells return to G0 even when encountering low levels of DNA damage, but the threshold of DNA damage required for returning to G0 increases as cells near S phase (20,36). This is consistent with the molecular model for APC/C-CDH1 inactivation, which is achieved in a sequential, stepwise manner, and also with the gradual degradation of CIP/KIP proteins (which inhibit CDK4/6 and CDK2) during the G1/S transition (20,43,108).

The impressive capacity of XRCC1-coordinated repair to correct SSBs means that levels of unrepaired SSBs in healthy cells are relatively low (11). However, SSB repair is lost in pathologies including cancer (109) and ataxia (110), and SSBs are induced by cancer therapies, including ionizing radiation, camptothecin, and PARP inhibitors (13,111). Furthermore, cancer cells often shorten G1 phase and expedite S phase entry by overexpressing oncogenes (112–115). This event may truncate the sensing of unrepaired SSBs in G1, promoting SSB to DSB conversion in S phase. Therefore, mechanisms to detect and signal SSBs between CP1/2 and CP3 may be most relevant in a pathological context. A long-sought method to measure patterns of SSBs at the genome-wide level was recently reported, which could have considerable impact for future research in this area (116).

## STOICHIOMETRIC CONTROL OF CDKs BY CYCLINS AND CDK INHIBITORS

Canonical models describing control of CDKs by cyclins and CDK inhibitors show that binding of a single molecule of the cognate cyclin to a single CDK molecule results in formation of an active cyclin-CDK complex, but the CDK is inhibited in the presence of a single CDK inhibitor molecule. This illustrates the molecular basis of 1:1 stoichiometric cyclin: CDK inhibitor control (3,5,117–122). To complement the structural studies that established this, the stoichiometric control was also characterised *in vivo* by the Meyer lab, who employed an elegant experimental setup using calibrated antibodies against cyclin D1 and p21 to specifically measure the cyclin D1: p21 ratio (19). Where this ratio is greater than one, CDK4 is active, while where the ratio is under one, CDK4 is inactive. In turn, this stoichiometric ratio is positively controlled by mitogens and negatively by DNA damage, to regulate S phase entry in an ultrasensitive manner (19,21,36).

Although the stoichiometric competition between cyclin D1 and p21 for regulation of CDK4 has been the attention of most research, not only p21 but all seven members of the INK4 and CIP/KIP families regulate CDK4. In addition, CDK2 is controlled by the stoichiometric competition between cyclin E and the CIP/KIP inhibitors during the G1/S transition (3,5). Furthermore, there are intricacies that mean that not all cyclin: CDK inhibitor complexes abide by the canonical rules for 1:1 stoichiometric competition. Therefore, a complete understanding requires a discussion of the functional interactions between CDK4/CDK2, their cognate cyclins, and the INK4 and CIP/KIP CDK inhibitors. Firstly, how are these CDKs activated?

CDK activation requires phosphorylation of a critical threonine in the activation segment (the T-loop), an inhibitory region of the CDK structure that occludes the active site (117,122). This phosphorylation is performed by the CDK-activating kinase (CAK), a trimeric complex of cyclin H-CDK7-Mat1 (5,117,122–124). For CDK1, cyclin binding precedes T-loop phosphorylation (5,124). For CDK2, T-loop phosphorylation of the CDK2 monomer initiates its activation, which is followed by binding of CDK2’s cognate cyclin, which stabilises the CAK-phosphorylated form of CDK2 and generates the active cyclin E/A-CDK2 holoenzyme (5,124). CDK4 is unusual in that its T-loop cannot be phosphorylated in monomeric nor cyclin-bound forms, and in that its direct binding to cyclin D is inefficient (5,120,125–127). Therefore, supplementary events are required to form the active CDK4 holoenzyme. CDK4 is additionally unusual in that it is an unstable CDK that requires the chaperone complex Hsp90–Cdc37 for folding. As such, nascent monomeric CDK4 molecules are immediately bound by Hsp90-Cdc37 (3,5,120,128,129).

### Fate of newly-synthesised CDK4 molecules

#### Control by the INK4 family

**Figure 5.**
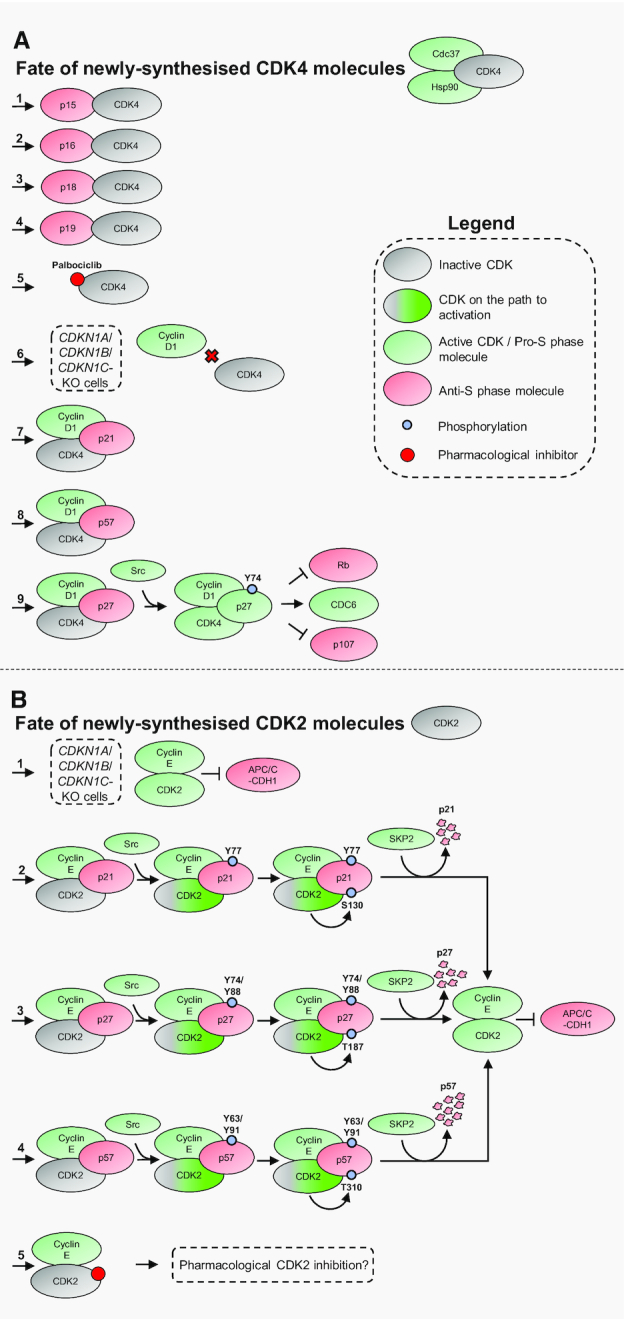
Stoichiometric control of CDKs by cyclins and CDK inhibitors. (**A**) Nine potential fates for nascent CDK4 molecules. **1–4**) INK4 family CDK inhibitors bind to inactive CDK4 monomers, sequestering CDK4 and preventing formation of the active CDK4 holoenzyme. **5)** The pharmacological CDK4/6 inhibitor Palbociclib mimics the INK4 family's mode of inhibition. **6**) p21/p27/p57 are redundantly required for cyclin D1-CDK4 assembly. **7–8**) Binding of p21 or p57 to cyclin D1 and CDK4 enables assembly of inactive cyclin D1-CDK4-p21/p57 complexes. **9**) Binding of p27 to cyclin D1 and CDK4 enables assembly of inactive cyclin D1-CDK4-p27 complexes. Subsequent phosphorylation of p27 at Y74 by Src, or other non-receptor tyrosine kinases, results in formation of the active cyclin D1-CDK4-p27 (Y74-P) holoenzyme. (**B**) Five potential fates for nascent CDK2 molecules. **1**) p21/p27/p57 are not required for assembly of cyclin E-CDK2 complexes. **2–4**) Binding of p21, p27 or p57 to cyclin E-CDK2 complexes inhibits CDK2. Subsequent phosphorylation of p21 at Y77, p27 at Y74/Y88, or p57 at Y63/Y91, by Src or other non-receptor tyrosine kinases, can stimulate cyclin E-CDK2 activity, leading to CDK2-mediated phosphorylation of p21/p27/p57 and the formation of phosphodegrons for SCF-SKP2. SCF-SKP2 directs the polyubiquitination and degradation of p21, p27, and p57, resulting in formation of cyclin E-CDK2 complexes that are active for phosphorylation and inhibition of APC/C-CDH1. **5**) Pharmacological CDK2 inhibitors bind and inhibit cyclin E-CDK2 complexes, although none have yet proven successful in the clinic.

p16, and its siblings in the INK4 family, binds CDK4 that has been freshly translated, outcompeting CDK4’s chaperone complex Hsp90–Cdc37. In this way, a single molecule of p16 sequesters a single molecule of CDK4, preventing oligomerisation into active complexes (5,119,121,128–130). Pioneering X-ray crystallography work lead to the discovery that this mechanism of action is mimicked by the CDK4/6 inhibitor Palbociclib, which is successfully used in the clinic for certain breast cancers (120,131) (Figure 5A).

#### Control by the CIP/KIP family

The mechanism for inhibition employed by the CIP/KIP family is considerably more nuanced, and has been best characterised for p21 and p27. Paradoxically, *CDKN1A*/*CDKN1B*/*CDKN1C* (p21/p27/p57) knockout cells cannot assemble cyclin D1-CDK4 complexes (127,132,133). Illuminating structural work has shown that this is because p21 and p27, which are intrinsically disordered as monomers, form helices upon binding to cyclin D1 and CDK4, spreading over both molecules to facilitate their interaction. That is, one molecule of either p21 or p27 binds one molecule of cyclin D1, and one of CDK4, to promote trimeric complex assembly (120). p21 and p27 also fill the role of enabling CAK-mediated CDK4 phosphorylation: binding of p21 or p27 induces structural rearrangements that liberate CDK4’s T-loop from its active site, facilitating phosphorylation of the T-loop by the CAK (120,134). Despite these activation mechanisms, the presence of either p21 or p27 (or p57) in cyclin D1-CDK4 complexes is potently inhibitory, both by preventing substrate binding to cyclin D1, and by distorting CDK4’s ATP-binding pocket (120,125) (Figure 5A).

An additional layer of complexity is provided by phosphorylation of p27 at Y74, performed by Src and other non-receptor tyrosine kinases, an event that was first thought to convert p27 from an inhibitor to a non-inhibitor of cyclin D1-CDK4 (125,126,135,136). However, in captivating findings, Guiley *et al.* have shown that cyclin D1-CDK4-p27 (Y74-P) complexes are not only relieved of inhibition, but actually display increased catalytic activity (compared to a recombinant cyclin D1-CDK4 dimer) toward some substrates (120). In this function, p27 is unique: p21 does not contain an equivalent residue for Y74 and cannot stimulate CDK4 activity in a similar manner (120). p27 is also phosphorylated at other tyrosines, such as Y88, but Y74 appears to be the predominant residue for regulation of CDK4 activity. Markedly, cyclin D1-CDK4 dimeric complexes are present at incredibly low levels in cells, suggesting that the physiologically-relevant CDK4 holoenzyme is a trimeric cyclin D1-CDK4-p27 (Y74-P) complex (120,137) (Figure 5A).

### Fate of newly-synthesised CDK2 molecules

#### Control by the CIP/KIP family

CIP/KIP family members, unlike INK4 members, additionally inhibit cyclin E/A-CDK2 complexes. CIP/KIP members fold by binding onto cyclin E/A followed by CDK2 in cyclin E/A-CDK2 complexes (5,138). The crystal structure for p27-cyclin A-CDK2 elucidated the inhibition mechanism employed by p27, showing that one molecule of p27 binds to a dimeric cyclin A-CDK2 complex, mimicking ATP in the catalytic cleft of CDK2 and preventing catalysis (118). Unlike their role in cyclin D1-CDK4 assembly, p21/p27/p57 are not required for cyclin E-CDK2 assembly (1,5,118) (Figure 5B).

However, like CDK4, tyrosine phosphorylation of p27 promotes CDK2 activity. Phosphorylation of p27 at Y74 or Y88 by Src (or other non-receptor tyrosine kinases) partially relieves p27’s inhibition of CDK2, enabling intra-assembly phosphorylation of p27—by the CDK2 that it is in complex with—at T187 (139–141). This phosphorylation forms a phosphodegron for SCF-SKP2, which consequently polyubiquitinates and promotes proteasomal degradation of p27, unleashing full CDK2 activity (92). Similarly, tyrosine phosphorylation of p21 at Y77, or p57 at Y63/Y91, by kinases like Src, stimulates CDK2-mediated phosphorylation (142). p21’s Src-mediated phosphorylation releases cyclin E-CDK2 from inhibitory interactions with p21, instead enabling non-inhibitory interactions that permit CDK2-mediated phosphorylation of p21, at S130. p57’s Src-mediated phosphorylation, although not explicitly proven experimentally, is also likely to result in relief of CDK2 activity, enabling CDK2-mediated phosphorylation of p57, at T310 (142). These CDK2-mediated modifications also form phosphodegrons for SCF-SKP2, and lead to p21/p57 degradation (61,93,142). A two-step procedure, started by Src, solves the riddle that poses how p21/p27/p57 degradation can be initiated when the protein that promotes p21/p27/p57 degradation (CDK2) is also the subject of their inhibition (Figure 5B).

### The cyclin D1-CDK4-p27 (Y74-P) holoenzyme *in vivo*

p27’s role in the physiologically-active CDK4 holoenzyme provides a pro-proliferative function of p27, to promote phosphorylation of CDK4 targets, including Rb, *in vivo* (120,125,126,135–137). However, p27-depleted or *CDKN1B*-knockout cells generally proliferate better than control cells, *CDKN1B*-knockout mice are characterised by increased growth and tumourigenesis, and levels of p27 are often reduced in cancer (95,143–145). This gives rise to two important questions.

Firstly, how do *CDKN1B*-deleted cells efficiently hyperphosphorylate Rb to sustain their excessive proliferation? In CDK4/6-inhibited cells, cyclin E-CDK2 activity can drive Rb hyperphosphorylation (41). Accordingly, it may be that the greatly enhanced CDK2 activity in p27-deficient cells (144) promotes Rb hyperphosphorylation in the absence of active CDK4. Notably, an increased reliance on CDK2 may be a therapeutic vulnerability, suggesting an opportunity for CDK2 inhibitors in cancers that delete *CDKN1B* (although, unlike CDK4/6 inhibitors, CDK2 inhibitors have not yet been successful in the clinic due to high toxicity (146)).

Secondly, would loss of p27 in cancer not universally reduce the activity of CDK4, a formidable oncogene with multiple targets? Although some tumours delete the *CDKN1B* gene, the major mechanism for downregulation of p27 in cancer is SCF-SKP2-mediated hyperdegradation (95,143). However, the affinity of SCF-SKP2 for T187-phosphorylated p27 is greatly enhanced if p27 is in a complex with cyclin E/A-CDK2 (but not cyclin D1-CDK4) (92,147–149). Moreover, the capacity for intra-assembly cyclin E/A-CDK2- (but not cyclin D1-CDK4-) mediated p27 phosphorylation to form the T187 phosphodegron (139–141) suggests that in cancer, it is the p27 complexed to cyclin E/A-CDK2—not cyclin D1-CDK4—that is the target for SCF-SKP2-dependent degradation. Specific degradation of p27 from cyclin E/A-CDK2 complexes would be a cunning oncogenic mechanism, enabling cancer cells to hyperactivate CDK2, while simultaneously retaining high CDK4 activity.

### Stoichiometric control of CDKs: conclusion

In conclusion, canonical rules state that one molecule of the cognate cyclin activates, while one molecule of CDK inhibitor inhibits, CDKs. Interactions between cyclin E, CDK2, and p21/p27/p57 abide by these rules, as does the INK4 family's inhibition of CDK4. p21/p57’s inhibition of cyclin D1-CDK4 largely abides by these rules, with the exception that p21/p57 promote cyclin D1-CDK4 complex assembly. However, cyclin D1-CDK4’s requirement of (Y74-phosphorylated) p27 for activity means that the cyclin D1-CDK4-p27 interaction decidedly neglects the canonical rules. Furthermore, the supplementary mechanisms that regulate CDKs—such as CAK- and SRC-dependent phosphorylation—highlight the additional complexities that impinge on the stoichiometric cyclin: CDK inhibitor competition (Figure 5). In the future, precise mass spectrometry-based quantification of the number of molecules of cyclins, CDKs and CDK inhibitors per cell would be an exciting direction of research for more deeply assessing how the stoichiometric competition works at the cellular level.

## SIX CONDITIONS FOR CANDIDATE INTEGRATORS OF MITOGENS AND DNA DAMAGE

We have discussed how certain molecules—such as cyclin D1—respond positively to mitogens and others—such as p21—respond positively to DNA damage, regulating the cyclin: CDK inhibitor stoichiometric balance depending on the relative mitogen: DNA damage load. However, given the critical role for the cyclin: CDK inhibitor balance in S phase entry, we propose that particularly potent regulation of this balance, consistent with its ultrasensitive nature (19,21), would require dedicated proteins that simultaneously respond, antagonistically, to both mitogens *and* DNA damage. Such signalling hubs would integrate mitogenic and DNA damage input, transmitting these signals to the stoichiometric cyclin: CDK inhibitor balance, and, consequently, the G1/S transition.

Proteins that are able to fulfil this role would satisfy the following six conditions: 1) regulation of the G1/S transition 2) regulation of cyclin D, cyclin E, or the CDK inhibitors 3) presence and activity in the mother G2, mother mitosis, daughter G0, or daughter G1 4) regulation by mitogens 5) regulation by DNA damage (opposite to the regulation by mitogens) 6) deregulation in cancer. For example, p53 fulfils conditions 1, 2, 3, 5 and 6, but not condition 4 (14), and therefore acts as a regulator, but not an integrator. Instead, we propose the following proteins that fulfil all six conditions.

### c-Myc

c-Myc is essential for S phase entry through its transcriptional stimulation of *CCND2* and transcriptional repression of *CDKN1A* and *CDKN2B* (which encodes p15). c-Myc performs these roles throughout the cell cycle, fulfilling conditions 1, 2 and 3 (7). c-Myc's proteasomal degradation is inhibited downstream of the mitogen-transducing MAPK signalling pathway. As a result, deprivation of mitogens causes degradation of c-Myc within minutes, while mitogen stimulation stabilises c-Myc (condition 4) (150,151). In addition, *MYC* (which encodes c-Myc) undergoes p53-dependent transcriptional repression upon DNA damage (condition 5). Many mechanisms have been suggested for this, including direct promoter binding by p53 (152). However, the most promising emerging mechanism depends on p53-mediated expression of the long noncoding RNA plasmacytoma variant 1 (*Pvt1b*), which is encoded 50 kb downstream of the *MYC* gene. *Pvt1b* accumulates near the *MYC* transcription start site upon DNA damage, inhibiting *MYC*’s expression (153,154). Finally, almost 30% of tumours express amplifications of *MYC* or its family members (155,156), fulfilling condition 6.

### Cyclin D1

Cyclin D1 is essential for S phase entry through its stimulation of CDK4/6 (condition 1), and automatically fulfils condition 2 in its capacity as a cyclin. Cyclin D1 can be active in the mother G2/M, daughter G0, and daughter G1, its protein levels peaking in G2/M phase (condition 3) (2,3,36,48). Mitogens induce the transcriptional stimulation of *CCND1* (157), as well as cyclin D1 post-transcriptional stimulation through cellular translation rates (condition 4) (48). In addition, DNA damage instigates rapid cyclin D1 degradation, mediated by the SCF ubiquitin ligase complex with the substrate-adapter F-box protein 31 (SCF-FBXO31) (condition 5) (106,158). The half-life of FBXO31 in undamaged cells is ∼2 h, due to its continuous degradation mediated by APC/C-CDH1 and APC/C-CDC20. However, ATM-mediated phosphorylation of FBXO31 inhibits its interaction with both CDH1 and CDC20, causing swift FBXO31 upregulation upon DNA damage (107). This permits degradation of cyclin D1, preventing S phase entry (106,158). In addition, the *CCND1* gene undergoes p53-dependent transcriptional repression following DNA damage (159), causing a slower and more sustained downregulation of cyclin D1 that maintains the quiescent state. Finally, in cancer, amplification of C*CND1* is among the most common genetic events (156) (condition 6).

Therefore, c-Myc and cyclin D1 are promising candidate integrators. Although these factors are well-established regulators of the cell cycle, the delicacy of the G1/S transition, and its susceptibility to DNA damage or hijack by cancer cells, suggests the requirement for multiple control mechanisms. The six conditions we set out can be used as a framework to identify new factors that integrate mitogenic and DNA damage signalling for control of the stoichiometric cyclin: CDK inhibitor balance at the G1/S transition, and will be an important line of future enquiry. Deregulation of these integrators in cancer would tilt the stoichiometric ratio of cyclins: CDK inhibitors constitutively in favour of the cyclins, enabling cancer cells to bypass requirement for mitogens and to ensure S phase entry even in the presence of DNA damage.

## CONCLUSION

Here, we discuss a model for the G1/S transition that unifies previous studies and theories. Central to the model is that mother cells play the predominant role in controlling the daughter cell S phase entry decision, through sensing of both mitogens and DNA damage. Mitogens and DNA damage are antagonistic signals, and must be integrated by cells to ensure high-fidelity S phase entry; we propose six defined conditions that would be fulfilled by candidate integrators. The role for these integrators is to transmit information from the mitogenic: DNA damage ratio to the stoichiometric cyclin: CDK inhibitor ratio, regulating CDK activity, the three commitment points for S phase entry (CP1, CP2 and CP3), and the G1/S transition. Although most cyclins and CDK inhibitors abide by canonical rules for 1:1 stoichiometric control of CDKs, a particularly interesting, paradigm-shifting finding is that the CDK inhibitor, p27, is required for formation of the active CDK4 holoenzyme *in vivo*, and inactivation of Rb in turn. Cells are mitogen-independent after Rb inactivation, which underpins CP1 and CP2, but can still reverse their decision to enter S phase—and return to quiescence—up until inactivation of APC/C-CDH1, which forms the molecular basis for CP3: DNA damage-resistant S phase entry. Sensing of unrepaired SSBs before CP3 is especially important, because replication of SSB-containing DNA induces DSBs, which can instigate genomic instability and oncogenesis (see main text for full references) (Figures 1–5).

The importance of factors that control CP1-3 and S phase entry is underscored by their enrichment for essential genes. This can be revealed through whole-genome CRISPR essentiality screens, so far performed in 769 cell lines with the goal of generating a Cancer Dependency Map (160–164) (Table 1. The website used for these data, provided by the Broad Institute, is: https://depmap.org/portal/). These screens elegantly highlight the relative contribution of different factors to the G1/S transition, as well as illuminating redundant, complementary mechanisms.

**Table 1.** Essentiality of core G1/S factors. Genome-wide CRISPR screens are at the centre of an effort to produce a Cancer Dependency Map (160–164). The website used for the data presented here, provided by the Broad Institute, is: https://depmap.org/portal/. The data represent the percentage of cell lines in which the stated gene is essential (i.e. where its CRISPR-mediated deletion is incompatible with viability)

G1/S factor (gene/protein)	Essentiality score (769 cell lines)	G1/S factor (gene/protein)	Essentiality score (769 cell lines)
*EGF*/EGF	0.0%	*PPP1CC*/PP1-γ catalytic subunit	8.3%
*EGFR*/EGFR	11.3%	*E2F1*/E2F1	17.3%
*MYC*/c-Myc	99.1%	*E2F2*/E2F2	0.4%
*CCND1*/Cyclin D1	79.8%	*E2F3*/E2F3A (+ E2F3B)	25.0%
*CCND2*/Cyclin D2	7.3%	*CCNE1*/Cyclin E1	7.8%
*CCND3*/Cyclin D3	15.9%	*CCNE2*/Cyclin E2	0.9%
*CDKN1A*/p21^Cip1^	0.0%	*CDK2*/CDK2	89.3%
*CDKN1B*/p27^Kip1^	0.4%	*SKP2*/SKP2	78.9%
*CDKN1C*/p57^Kip2^	0.1%	*FBXO5*/EMI1	99.2%
*CDKN2A*/p16^INK4A^ + p14^ARF^	0.5%	*FZR1*/CDH1	15.9%
*CDKN2B*/p15^INK4B^	0.1%	*XRCC1*/XRCC1	43.4%
*CDKN2C*/p18^INK4C^	0.0%	*ATM*/ATM	1.2%
*CDKN2D*/p19^INK4D^	0.0%	*PARP1*/PARP1	0.9%
*CDK4*/CDK4	46.8%	*MDM2*/MDM2	27.4%
*CDK6*/CDK6	52.1%	*TP53*/p53	4.8%
*SRC*/Src	2.2%	*CCNA1*/Cyclin A1	0.0%
*RB1*/Rb	0.0%	*CCNA2*/Cyclin A2	99.9%
*PPP1CA*/PP1-α catalytic subunit	69.6%	*RRM2*/RRM2	100.0%
*PPP1CB*/PP1-β catalytic subunit	79.9%		

Consistent with essential roles for proliferation, proteins driving the G1/S transition are the targets for promising cancer therapies. For example, small molecule inhibitors of SKP2 (165) and c-Myc (166) show promise in pre-clinical studies, and CDK4/6 inhibitors are clinically approved for use in certain breast cancers (4,131,167). In the future, opportunities for proteolysis-targeting chimeras (PROTACs) (168) or molecular glues degraders (169) will facilitate the targeting of newly-identified G1/S factors, as well as previously undruggable proteins.
